# Supplementary material for: Saccharomyces boulardii (CNCM I-745) improves intestinal damage in sepsis by remodeling intestinal flora structure
Source: Front Cell Infect Microbiol. 2025 Jul 31;15:1602792. doi: 10.3389/fcimb.2025.1602792 (PMC12350329; doi:10.3389/fcimb.2025.1602792)
Supplement: Supplementary file 1 [file DataSheet1.zip › summary/summary/7_Differential_analysis/1_diff_abundance/7_Species/Group.DW4_vs_BW4/Group.DW4_vs_BW4_diff_barplot.pdf]

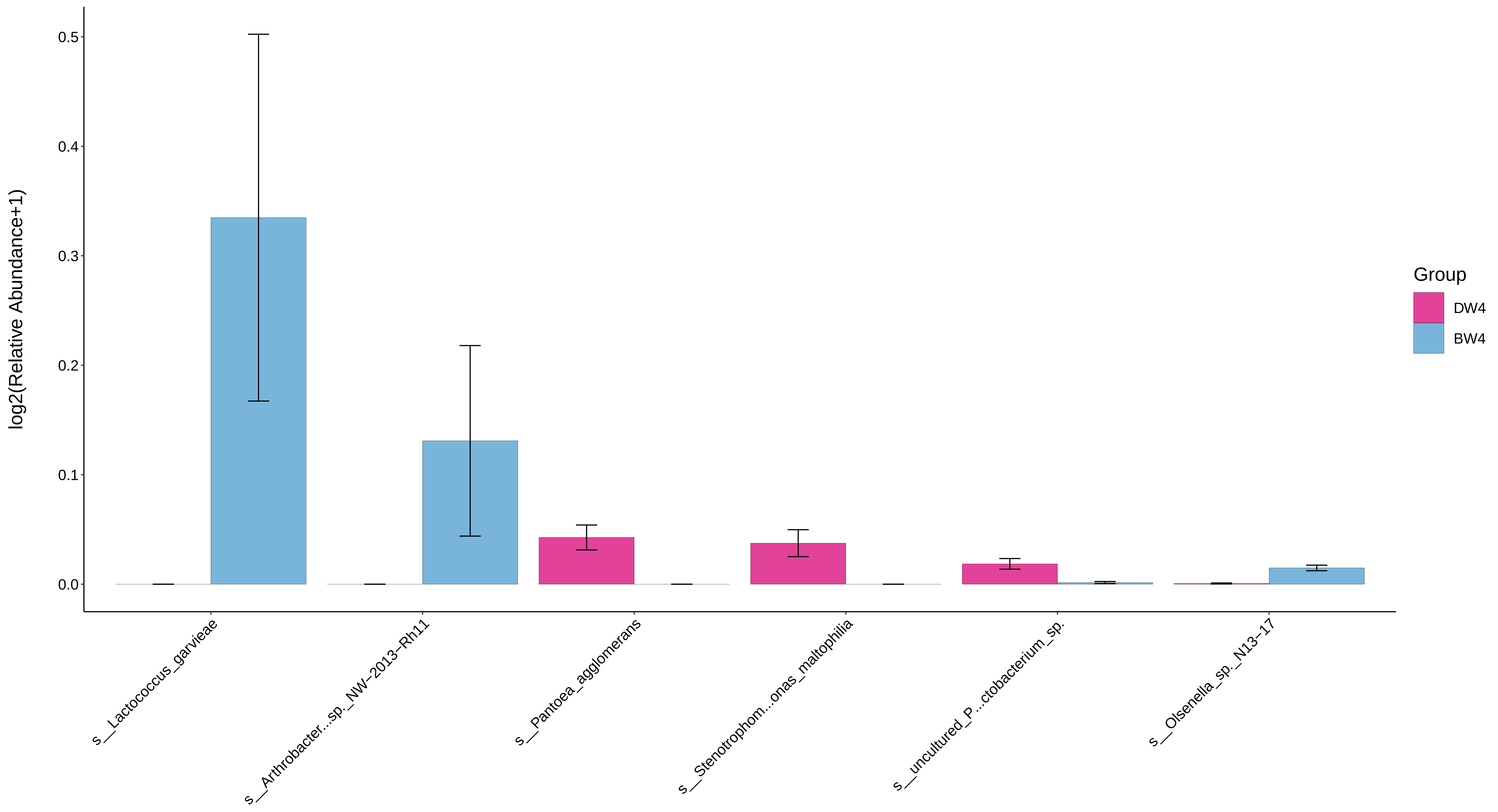

log2(Relative Abundance+1)
0.5
0.4
0.3
0.2
0.1
0.0
Group
DW4
BW4
s__Lactococcus_garvieae
s__Arthrobacter...sp._NW−2013−Rh11
s__Pantoea_agglomerans
s__Stenotrophom...onas_maltophilia
s__uncultured_P...ctobacterium_sp.
s__Olsenella_sp._N13−17
